# Negotiating Left-Hand and Right-Hand Bends: A Motorcycle Simulator Study to Investigate Experiential and Behaviour Differences Across Rider Groups

**DOI:** 10.1371/journal.pone.0029978

**Published:** 2012-01-11

**Authors:** Elizabeth Crundall, David Crundall, Alex W. Stedmon

**Affiliations:** 1 Accident Research Unit, School of Psychology, University of Nottingham, Nottingham, United Kingdom; 2 Centre for Motorcycle Ergonomics & Rider Human Factors, Faculty of Engineering, University of Nottingham, Nottingham, United Kingdom; The University of Western Ontario, Canada

## Abstract

Why do motorcyclists crash on bends? To address this question we examined the riding styles of three groups of motorcyclists on a motorcycle simulator. Novice, experienced and advanced motorcyclists navigated a series of combined left and right bends while their speed and lane position were recorded. Each rider encountered an unexpected hazard on both a left- and right-hand bend section. Upon seeing the hazards, all riders decreased their speed before steering to avoid the hazard. Experienced riders tended to follow more of a racing line through the bends, which resulted in them having to make the most severe changes to their position to avoid a collision. Advanced riders adopted the safest road positions, choosing a position which offered greater visibility through the bends. As a result, they did not need to alter their road position in response to the hazard. Novice riders adopted similar road positions to experienced riders on the left-hand bends, but their road positions were more similar to advanced riders on right-hand bends, suggesting that they were more aware of the risks associated with right bends. Novice riders also adopted a safer position on post-hazard bends whilst the experienced riders failed to alter their behaviour even though they had performed the greatest evasive manoeuvre in response to the hazards. Advanced riders did not need to alter their position as their approach to the bends was already optimal. The results suggest that non-advanced riders were more likely to choose an inappropriate lane position than an inappropriate speed when entering a bend. Furthermore, the findings support the theory that expertise is achieved as a result of relearning, with advanced training overriding ‘bad habits’ gained through experience alone.

## Introduction

Motorcyclists are grossly over-represented in accident statistics. As of June 2010, motorcycles constituted less than 1% of the total vehicle miles on UK roads, but accounted for 21% of all UK road fatalities [1]–[3]. A recent report illustrated that sixty-five per cent of such motorcycle fatalities occur in rural areas [4]. Clarke et al. [5], [6] noted that a large proportion of these accidents occur because the motorcyclist loses control on a bend or corner. These accidents are usually regarded as the fault of the motorcyclist, and often do not involve any other traffic. Loss of control accidents on bends are associated with riding for pleasure, and are also related to inexperience. Riders who have not had a license for long (or who have returned to motorcycling after a number of years) as well as riders who hold a provisional license, are more likely to be involved in a loss of control accident on a bend [5], [6]. The accidents tend to be a result of the motorcyclist running wide of the curve due to inappropriate speed or under-steering through the bend.

There is also evidence to suggest that, in general, crashes are more likely to happen on sharp bends than on gentle bends [7]–[10]. Furthermore, there are studies that indicate that left-hand bends in the UK are more dangerous than right-hand bends [11], [12]. This is thought to be due to a greater difficultly in perceiving curvature when riding on the inside of the bend, although this problem can be ameliorated by riding closer to the centre line of the road on a left-hand bend.

While accidents involving all types of traffic might be more prevalent on left-hand bends, right-hand bends might still pose a specific danger for motorcyclists. In an analysis of motorcycle accidents in Scotland [13] 9% were associated with right-hand bends, which was only slightly less than accidents involving left-hand bends (11.4%). Stewart and Cudworth [12] suggest that some right-hand bend accidents occur on ‘S bends’, where the accident terminates on a right-hand bend but is actually initiated on an immediately preceding left-hand bend. However, it remains a possibility that some right-hand bend accidents might occur as a result of a perceptual error in judging the acuteness of the bend. Considering the risky relationship between motorcycles and bends, we believe it is important that more research be focussed upon identifying the problems of navigating curves and the successful strategies employed by the safest riders.

Recently, the introduction of simulators has provided transport researchers with a useful tool for investigating the behaviour of road users in a safe environment whilst still retaining an acceptable level of ecological validity [14]. Furthermore, simulations offer a means of manipulating the environment in a controlled way that is not possible in the real world. Thus it is possible to assess how different road users with varying levels of skill and training cope with the same situation. For instance, Crundall, Andrews, van Loon and Chapman [15] compared trained and untrained car drivers on their approach to a series of highly controlled virtual hazards, and noted the positive impact of training on speed and braking signatures on the approach to the hazards (see also [16], [17]).

Driving simulators have also been used to assess the behavioural effectiveness of road engineering factors upon speed choice of drivers when negotiating bends. Drivers approaching bends demonstrated improved speed adaptation if the curve radius was highlighted, either implicitly (e.g. with hazard marker posts or chevrons) or explicitly (e.g. with an advisory speed sign or flashing warning) [18]. While such highly controlled yet ecologically valid studies are still limited within the car driving domain, as far as the authors are aware they are non-existent in the motorcycle literature. There is a limited amount of research on motorcycle simulators which has focussed upon detection and avoidance of hazards, though levels of control and manipulation have not reached those of comparable studies with car simulators [19]–[22].

### Rationale

The current study used a new motorcycle simulator (MotorcycleSim) to investigate rider behaviour in bends. MotorcycleSim was developed at the University of Nottingham and uses a full motorcycle interface linked to a large screen presentation of specially-modified “STISIM-Drive” software [23]. Thus the simulator retains the functional fidelity of a real motorcycle whilst providing complete control over the development of road geometry, and the ability to record a wide variety of measures at a high sample rate.

Given that accidents on bends are linked with inexperience, the aim of the study was to compare riders of varying skill and experience in order to assess the problems associated with navigating a series of left and right bends, and identify the potential strategies that the safer riders might adopt. For instance one might expect that experienced or advanced riders crash less frequently on bends as a result of better road positioning as well as a more appropriate choice of speed. The following experiments were therefore designed to compare novice, experienced and advanced riders in order to investigate if experience and advanced training offer accumulative, or different, benefits when negotiating bends, especially when faced with an unexpected hazard hidden around a bend, such as a broken-down vehicle. Advanced riders were defined as riders who had passed an advanced motorcycle test following training provided by the Institute of Advanced Motorists (IAM) in the last 3 years. This advanced training course focuses on a system of motorcycle control as a way of approaching and negotiating potential hazards in a methodical, safe manner and encompasses five factors of safe riding: Information, Position, Speed, Gear and Acceleration or the ‘IPSGA’ system of motorcycle control [24].

There are two key influences on how a rider might approach a bend. The first is progression: In order to make the fastest progression through a bend, a rider might adopt what is known as the ‘racing line’, moving from the outside edge of a curve to the inside edge as they approach the apex. After passing the apex the rider then moves back out towards the outside edge when exiting the curve. The intention of this manoeuvre is to minimise the travel time through the curve by optimising the distance travelled with the speed that can be successfully maintained. The other influence is safety: In the UK, when negotiating a left hand bend, a rider might adopt a position closer to the centre line in order to gain the best visibility throughout the bend. Conversely, on a right hand bend, a rider might adopt a position closer to the left-hand side of the road to optimize visibility throughout the bend. Since two key aspects of training are safety and progression, we might expect advanced riders to only take the racing line after they have obtained a sufficient view of what is around the bend.

The DSA recognise the importance of positioning in bends, and this is included in the road test. Novice riders preparing for the DSA test are formally taught to change their position to the left to increase visibility on a right hand bend, and to maintain a dominant (centre of lane) position on a left hand bend, balancing visibility with the need to avoid oncoming traffic which is close to the centre line. Furthermore, novices are taught to consider the physics of the motorcycle when navigating a bend: Given that riders typically lean towards oncoming traffic on a right-hand bend, and towards road furniture on a left-hand bend, taking a severe progressive line could mean that there is more danger of the rider coming into contact with other objects, even if the wheels of the motorcycle are within the correct lane. However, despite their recent training, novices are more likely to be involved in accidents on bends, so we might expect this group to choose sub-optimal positioning and speeds around bends compared to the experienced and advanced riders. For instance, over-confidence might lead them to take a more pronounced racing line which eschews safety concerns. Alternatively, it is plausible that they try to optimise visibility but still under-estimate speed.

In the following experiments, we recorded speed and lane position of riders navigating two sets of left and right bends on two laps of a virtual riding scenario. In all bends the ground at each side of the carriageway was banked and populated with trees to prevent riders from seeing beyond the apex of the curve. This control allowed for both the ‘racing line’ and the ‘visibility line’ to be equally plausible riding options.

In addition to monitoring riding style on empty bends, riders encountered two hazards on the second lap. The first hazard was positioned on the penultimate left bend and the second hazard was positioned on the penultimate right bend. This provided an opportunity to assess whether the behaviour that was adopted in the earlier curves had an impact on the extent of the avoidance behaviour that was required upon meeting the hazard. The left-bend hazard was a broken-down car on the left-hand verge, hidden around the apex of the curve. The right-bend hazard was an oncoming vehicle in the contra-flow lane, driving near the centre line of the road. Both hazards were designed to cause problems for riders who favoured a severe racing line at the expense of visibility.

Finally, measures taken from the navigation of the final left and right bends (post-hazard) provided an opportunity to assess whether the riders immediately changed their approach to the curve as a result of encountering the hazard on the previous bend. It was predicted that advanced riders would adopt a speed and lane position that was commensurate with the dangers posed by a blind bend (i.e. slower and more towards the centre line in the left-hand bends, or more towards the left in the right-hand bends), and as such should require less modification to their riding style when the hazard was spotted. It was predicted that there would be more similarities between the experienced riders and the advanced riders in terms of riding style, than between the novices and advanced riders. We believed the novices were most likely to favour progression over safety adopting an early racing line and inappropriate speed. Thus novices should be required to make greater changes to their position and speed on encountering the hazard, but might be more likely to amend their behaviour on the subsequent bend.

## Methods

### Ethics Statement

Ethical approval for the study was sought on 12th August 2010. The application was reviewed in accordance with the University of Nottingham Faculty of Engineering Ethics Committee protocols and approval was granted on 24th August 2010. All participants gave informed written consent before taking part in the experiment.

### Participants

Sixty two participants were recruited for the study and reimbursed for their time. The participants were all members of the general public and were recruited through local and national adverts, through rider training schools, local motorcycle meetings and the national IAM newsletter. Participants were pre-screened for excessive driving experience so that anyone with a typical annual mileage in excess of 20,000 miles per annum or who held any specialist driving licence (e.g. public service vehicle, light or heavy goods vehicle) was excluded from the study. All participants held at least a provisional UK motorcycle license, had normal or corrected to normal vision and did not suffer from migraines, epilepsy or motion sickness. From the sample, 61 participants completed the study and their data were used for analysis (one participant withdrew with simulator sickness symptoms). Of the participants, 20 were novice riders (having taken or were preparing to take the standard Driving Standards Agency (DSA) motorcycle test within the last 12 months); 21 were experienced riders (who had over 3 years riding experience since passing their motorcycle test, but had no further training) and 20 were advanced riders (who had passed their IAM advanced riding test in the last 3 years). Novice riders were younger than the other 2 groups (mean age = 26.5 years; SD = 8.2 years), while the advanced riders (mean age = 47.4 years; SD = 9.2 years) were slightly older than the experienced riders (mean age = 40.6 years; SD = 9.3 years). Experienced riders and advanced riders were matched for overall riding experience (experienced riders mean experience = 15.6 years; SD = 9.9 years, and advanced riders mean experience = 16.6 years; SD = 11.9 years). There was no significant difference between the 3 groups in terms of the amount of hours spent riding per week (p>0.05).

### Design

Riders rode two laps of a simulated riding scenario, and encountered a set of four left-hand bends and a set of four right-hand bends on each lap. Left-hand bends and right hand bends were analysed independently, but were identical in terms of experimental design. Two sets of analyses were performed on each set of bends. The first set of analyses was concerned with the appearance of a hazard on the third bend of the second lap (which we shall refer to as L2B3: Lap 2, Bend 3). The design adopted for this analysis was a 3×7 mixed design, with measures from the three rider groups (novice, experienced and advanced) compared across seven sections of the hazard bend. Each section of the bend was 100 ft long (measured along the centre line of the road). The middle 5 sections each comprised a 14.3 degree section of the curve, and the 1st and 7th sections comprised the entry spiral and exit spiral respectively. Spirals are standard terms in geometry for sections of road that connect straight and curved sections. While curves have a constant curvature, spirals linearly increase in curvature from 0 (when the road is straight) until they join up with the full curve. This provides a gradual transition from straights to curves (and vice versa) and allows the road user to prepare for the full bend.

Mean speed, mean lateral position and variance of lateral position were calculated for the seven different sections in order to explore whether these measures changed as riders progressed through the curve and encountered the hazard. Both the left-bend hazard and the right-bend hazard were located between the 5th and 6th sections of the bend, and were visible from section 3. The left bend hazard was a stationary, broken-down car, but the right bend hazard was a moving vehicle, which continued around the curve in the contra-flow lane, driving near the centre line.

The second set of analyses required a more complex design. A 2×2×3×7 mixed design aimed to compare measures on two bends; one preceding and one following each hazard (bend 2 and bend 4), across both laps (Lap 1 and Lap 2). Any differences between the measures on the second and fourth bends of Lap 2 (L2B2 vs. L2B4) could be due to participants encountering the hazard in the intervening bend (L2B3). However, no hazard occurred between bends 2 and 4 on Lap 1 (L1B2 and L1B4), so these bends provided baseline data to compare against. The other two factors were the three rider groups and the seven curve sections as used in the analysis of the hazard bend. The first bend was excluded from all analyses, since this bend was immediately preceded by a straight road while all other bends were preceded by another bend. Therefore, the road geometry of, and immediately prior to, each analysed bend was identical. Average speed (mph), lateral position (ft) and variance of lateral position (ft^2^) were calculated for the seven curve sections in order to explore if any effects were isolated to particular parts of the bends (e.g. the apex or the spiral).

The reason for this choice of design was to (a) identify if there were any experiential differences in riders' immediate responses to the hazard, (b) identify if there were any differences in the way rider groups approached bends prior to encountering the hazard, and (c) identify if riders modified their behaviour on bends after experiencing the hazard (i.e. whether there were any differences between pre- and post-hazard bends on Lap 2).

A comparison of Lap 2 Bend 2 (L2B2) with Lap 2 Bend 4 (L2B4), and a comparison of L1B4 with L2B4 would reflect any effects of experiencing the hazard. However, any differences between these bends might also include familiarity or practice effects. A comparison of L1B2 with L1B4 provided a baseline estimate of immediate practice within a lap, and a comparison of L1B2 with L2B2 provided an estimate of familiarity and practice from Lap 1 to Lap 2. Therefore, any interactions between bend and lap should help to isolate any effects of experiencing the hazard.

### Apparatus and Stimuli

The main apparatus for the study was the MotorcycleSim simulator which consists of a full size motorcycle (Triumph Daytona 675), two pairs of pneumatic actuators and user input controls. The simulator was used in static mode throughout the study with the pneumatic actuators pressurised to stabilise the motorcycle and rider. The user input controls provided data to the “STISIM-Drive” software, which used this data to provide appropriate visual feedback via a simulated visual environment. The standard motorcycle controls (e.g. throttle, gears, braking input and steering angle) were used to place the rider in the correct location and control their interaction within the scenario. The “STISIM-Drive” software was operated with a tilting horizon and pitching action to support the riding experience of leaning into bends and braking and acceleration effects [23]. Speed and lateral position of the rider were recorded at a frequency of 10Hz.

The scenario was presented on a large flat-screen (285cm×228cm with a resolution of 1280×1024) at a distance of 190cm in front of the rider. A speedometer, tachometer, gear selection and view of the road behind the rider were presented at the bottom of the screen. Auditory feedback of engine noise (based on current rpm) was also provided using surround sound speakers.

A bespoke scenario was developed for the study which covered a total distance of 101,000 ft (30,793m). The scenario comprised two laps of a simulated route which included a mixture of urban, suburban and rural roads, with riders spending approximately an equal amount of time in 40 mph speed limit zones (i.e. urban and suburban) and 60 mph zones (i.e. rural). Within the scenario, a number of sub-scenarios were designed to investigate different aspects of riding behaviour [25]. Of interest in the current paper are the left-hand and right-hand S-bends, embedded in one of the rural sections of the riding scenario.

**Figure 1:**
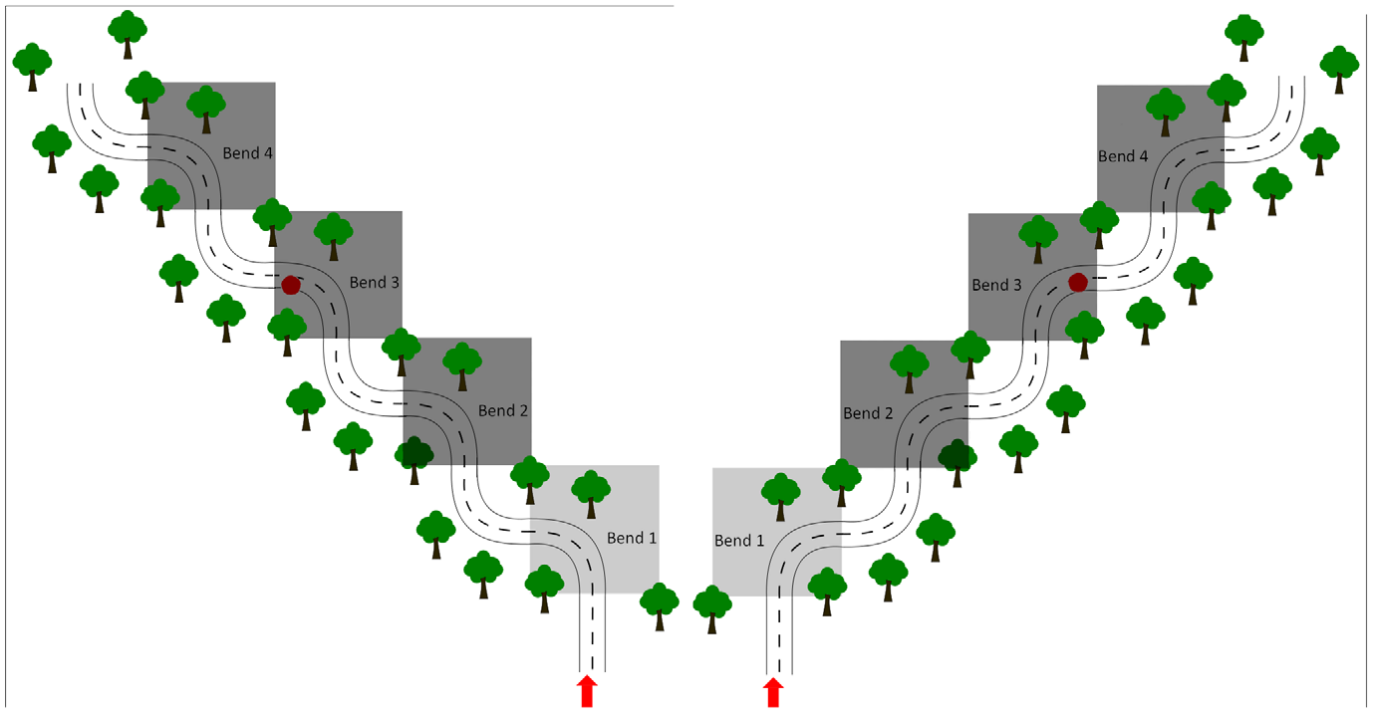
Schematic depictions of the road geometry of left-hand S-bends (left panel) and right-hand S-bends (right panel). The dark grey boxes represent analysed bends (i.e. bends 2, 3 and 4). The dots in Bend 3 denote the positions of the hazards. Entry point into the S-bends and direction of travel are represented by arrows.

The S-bends comprised eight pairs of opposing left- and right-hand bends designed to mimic riding a series of bends in a rural setting with a speed limit of 60 mph. There was a set of 4 left-hand bends, each immediately followed by an opposing right-hand bend. This was followed by a 7400 ft section of straight road, then a set of 4 right-hand bends, each immediately followed by an opposing left-hand bend. Left-hand bend data were collected from the first set of bends, and right-hand bend data were collected from the second set (i.e. the relative opposing bends in each set of bends were not analysed). Data from the first bend in each set were excluded from any analyses, so that only bends 2, 3 and 4 were analysed. This ensured that the road geometry was identical immediately before each analysed bend, since approach position was likely to be affected by the preceding manoeuvre. Maps of the left-hand S-bends and the right-hand S-bends are shown in Figure 1.

The bends were 700 ft long and comprised a 500 ft section with constant curvature of 0.0025 (equivalent to the reciprocal of the radius in feet) preceded by a 100 ft entry spiral (in which the curvature of the road increased linearly from 0 to 0.0025) and followed by a 100 ft exit spiral (in which the curvature of the road decreased linearly from 0.0025 to 0). Both sets of S-bends were preceded by a standard UK warning sign for bends ahead, positioned 600 ft before the first bend.

Trees were positioned either side of the road from 1100 ft before the bends to approximately 3000 ft after the bends. In addition, an embankment on either side of the road was designed to prevent the rider from ‘seeing through’ the bends. However it was possible to ascertain the road layout from the tree-line beyond the vanishing point of the road.

**Figure 2:**
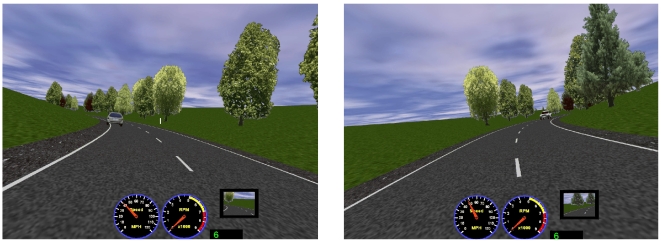
Rider view of vehicle hazard on the left-hand bend (left panel) and on the right-hand bend (right panel).

On the second lap, a hazard appeared on bend 3 (L2B3) in both the sequence of left-hand bends and the sequence of right-hand bends. On the left L2B3 a stationary car was positioned 150 ft beyond the apex of the bend (Figure 2: left panel). If the rider was positioned close to the centre line as they progressed around the left-hand bend, the car was visible at a distance of 289 ft. However, if the rider was positioned nearer to the kerb, the car only became visible from 263 ft away.

For the right-bend hazard a car travelling in the opposite direction was positioned legally but close to the centre line of the road. When the motorcyclist reached a point 150 ft prior to the apex of the bend, the car was initially positioned 150 ft beyond the apex of the bend, and travelled at a constant speed of 6.8 mph (Figure 2: right panel). A slow speed was chosen so that the vehicle was encountered by each participant approximately at the same point on the road. If the rider was positioned to the left hand side of the road upon entering the bend, the car became visible when the rider was approximately 300 ft from the hazard. No other vehicles appeared in this part of the route, although traffic did appear in other sections of the main riding scenario.

### Procedure

Participants conducted two practice sessions on MotorcycleSim to familiarise them with the simulator controls (e.g. steering, throttle response, gears, and braking inputs), an emergency stop (to assess the braking potential of the simulator) and overtaking manoeuvres around the slow moving vehicles. The two practice sessions lasted between 8 to 10 minutes in total. Throughout the experiment, participants wore a helmet whilst on the simulator so that their field of view would be similar to that experienced on a real motorcycle.

Participants then completed the main riding scenario. They were instructed that the route would take 20 to 25 minutes to complete and that it would repeat itself half way through. Participants were also instructed to ride as they would in the real world. If participants left the main carriageway (by more than 1 ft on either side of the road) an accident was registered, the scenario paused and they were placed back on the road to continue the simulated route from that point. An allowance of 1 ft meant that riders could pull over to the road side if necessary without it being recorded as an accident. Measures of simulator sickness symptoms were recorded in between the practice and testing sessions. Finally, participants were fully debriefed on the purpose of the study and paid for their time.

## Results

The results for the left-hand S-bends are reported first, followed by the results of the right-hand bends. Each series of bends was analysed in two ways. First participants' immediate responses to the hazard on L2B3 were analysed. Mean speed, mean lateral position and variance of lateral position were calculated for the seven curve sections in order to explore whether these measures changed as riders progressed through the curve and encountered the hazard (which was positioned between the 5th and 6th sections, and visible from the 3^rd^ section onwards).

The second analysis compared the non-hazard bends across the factors of Lap and Bend number (L1B2, L1B4, L2B2 and L2B4). This analysis revealed any differences in how the riders generally approached the bends, and whether they changed their behaviour following their encounter with the hazard. All interactions were explored with repeated interaction contrasts and analysis of simple main effects. Within group main effects were explored with repeated contrasts, while post-hoc between group main effects were analysed using Scheffé tests.

### Responses to the left-hand hazard bend (L2B3)

**Figure 3:**
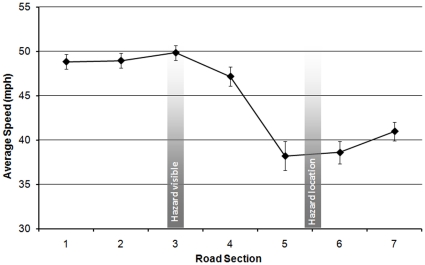
Average speed for left-hand hazard bend sections (L2B3).

A series of 3×7 ANOVAs were conducted on data for speed, lateral position, and the variance of lateral position comparing the measures for each group across the seven sections of the curve. Analysis of average speed revealed a significant effect of curve section (F(6,348) = 59.603; MSe = 27.411; *p*<0.001). Repeated contrasts indicated that riders significantly increased their speed from an average of 49.0 mph to 49.8 mph between sections 2 and 3 then significantly decreased their speed to an average of 47.2 mph on section 4. This corresponds with the hazard becoming visible in section 3. Riders dropped their speed more dramatically to 38.2 mph on section 5 then continued at a similar speed before significantly increasing their speed to an average of 41.0 mph on section 7 (all at *p*<0.05). These findings are illustrated in Figure 3. There was no group effect.

**Figure 4:**
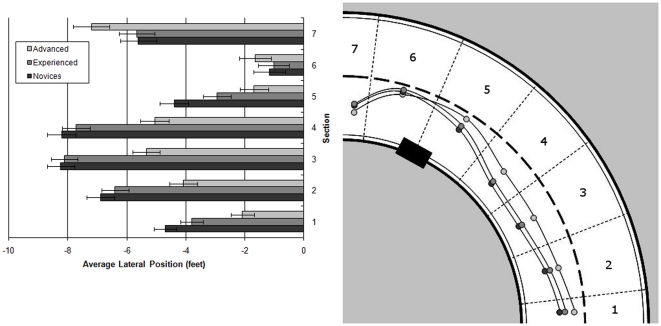
Lateral position across the seven bend sections for the rider groups. The left panel represents this as bars with standard errors added, while the right panel is a schematised plan view of the data.

Mean lateral position of the motorcycle produced a measure between −12 ft and +12 ft where zero was the centre line of the road and increasingly negative numbers reflect positions further to the left of the lane, with a score of −12 reflecting the left edge of the road. Any positive score of mean lateral position reflects an incursion into the contra-flow lane. A significant main effect was observed for rider group upon lane position (F(2,58) = 8.802; MSe = 1.813; *p*<0.001). Scheffé tests revealed that advanced riders rode significantly closer to the centre line (mean = −3.9 ft) than both experienced (mean = −5.093 ft; *p*<0.05) and novice riders (mean = −5.604 ft; *p*<0.01). However, experienced and novice riders did not differ significantly (*p* = 0.482). A main effect of curve section was also observed (F(6,348) = 86.002; MSe = 3.647; *p*<0.001) as well as an interaction between rider group and curve section (F(12,348) = 6.271; MSe = 3.647; *p*<0.001). Repeated contrasts revealed that this interaction was significant only between sections 4 and 5 (*p*<0.05) and between sections 5 and 6 (*p*<0.01). Through sections 1 to 4, despite all riders moving across to the left edge of the road, the advanced riders stayed significantly closer to the centre line than both the novice and experienced riders (*p*<0.01, as indicated by simple main effects analysis comparing rider groups at each level of road section). However, when the riders reached section 5, all groups moved back towards the centre line, but due to their early positioning the advanced riders were still significantly closer to the centre than the novice riders (*p*<0.01). The experienced riders made an extreme shift towards the centre line in section 5 so that their lateral position was no longer different to that of the advanced riders (though they were still statistically indistinguishable from the novice riders also). Between sections 5 and 6, the advanced riders maintained their central road position whilst the other two rider groups continued to move towards the centre line. Finally, all three groups moved over to the left in the exit spiral (section 7) in preparation for the upcoming right-hand bend. These findings are illustrated in Figure 4.

The right panel summarises the results in a schematic fashion. Essentially the advanced riders took a safer approach closer to the centre line, moving out slightly in section 5 to ensure avoidance of the hazard. The novice and experienced riders had to make more pronounced shifts towards the centre line and they achieved a safe position significantly later than the advanced riders. The novice and experience riders exhibited swerving behaviour in response to the hazard whilst the advanced riders were able to make smaller changes to their road position and in more time due to being able to observe the hazard earlier.

**Figure 5:**
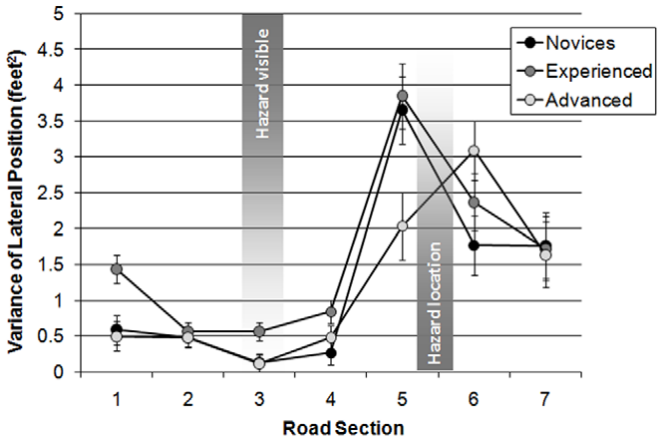
Variance of lateral position across the seven bend sections for the rider groups (with standard error bars added).

The variance of lateral position was also analysed to assess the extent of any shifts in lane position across the seven sections. There was a significant main effect of rider group (F(2,58) = 3.407; MSe = 0.339; *p*<0.05), a main effect of curve section (F(6,348) = 38.719; MSe = 1.939; *p*<0.001), and a significant interaction between rider group and curve section (F(12,348) = 2.536; MSe = 1.939; *p*<0.01). The interaction is illustrated in Figure 5.

The main effect of rider group was due to experienced riders (mean = 1.62 ft^2^) displaying more lateral movement than the advanced riders (mean = 1.19 ft^2^), although this was not statistically significant in the Scheffé tests (*p* = 0.069). However, neither the experienced riders nor the advanced riders differed significantly from the novices (mean = 1.24 ft^2^). In general, there were significant differences in the amount of lateral movement between each of the seven sections (*p*<0.05) except between sections 6 and 7 (*p* = 0.096).

Repeated contrasts revealed that the interaction between rider group and section was significant at the beginning of the bend (between sections 1 and 2, *p*<0.05) and near the hazard (between sections 4 and 5, *p*<0.05; and between sections 5 and 6, *p*<0.01). Simple main effects analysis and Scheffé tests revealed that during section 1, the experienced riders displayed more lateral movement than both novice (*p*<0.05) and advanced riders (*p*<0.01). All 3 groups of riders made an increase in lateral movement between sections 4 and 5 in response to the hazard (*p*<0.001), but the advanced riders displayed less lateral movement than the experienced riders (*p*<0.05) and demonstrated a near-significant difference when compared to the novice riders (*p* = 0.059). This latter finding supports the suggestion that the advanced riders had prepared their lane position for passing the hazard sooner than the other two groups, and therefore needed a significantly smaller correction to their lane position. The novice and experienced riders decreased lateral movement between sections 5 and 6, although this only approached statistical significance for experienced riders (for novices *p*<0.01; for experienced *p* = 0.053). To summarise, the interaction results arise from the experienced riders making more lateral movement at the beginning of the bend and from novices and experienced riders making more lateral movement in response to the hazard.

### Responses to the left-hand non-hazard bends

A series of 3×2×2×7 ANOVAs were conducted on data for speed, lateral position and the variance of lateral position, comparing the three rider groups across Lap 1 and Lap 2, and across the second and fourth bend (L1B2, L1B4, L2B2 and L2B4), for all seven sections of each curve. It should be noted that the hazard occurred on the 3rd bend of Lap 2 (L2B3), so only L2B4 is considered to be post-hazard, while the other three bends acted as different control conditions to compare against.

**Figure 6:**
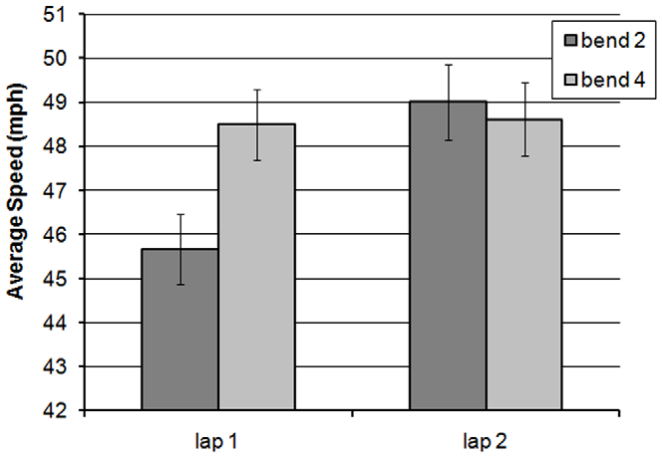
Average speed of riders on the 2nd and 4th left-hand bends across the two laps (with standard error bars added).

Analysis of the riders' mean speed revealed a significant main effect of bend (F(1,58) = 7.633; MSe = 82.095; *p*<0.01), a significant main effect of lap (F(1,58) = 8.589; MSe = 147.813; *p*<0.01), and an interaction between the two (F(1,58) = 15.177; MSe = 72.914; *p*<0.001). Simple main effects showed that on bend 2, riders were significantly faster during Lap 2 than they were on Lap 1(L2B2>L1B2; F(1,60) = 26.741; MSe = 12.756; *p*<0.001). We suggest that this represents a familiarity effect, with riders feeling more comfortable with higher speeds having already navigated the bend once before. However, there was no significant difference between Lap 1 and Lap 2 for bend 4 (*p* = 0.850). The familiarity effect was possibly overridden by the appearance of the hazard on L2B3. Furthermore, riders increased their speed between L1B2 and L1B4 (F(1,60) = 20.834; MSe = 11.468; *p*<0.001), but did not display this increase in speed on Lap 2 (*p* = 0.512). Instead of an increase, riders decreased their speed slightly on Lap 2 Bend 4. Again, this suggests that, on the first Lap, riders became more comfortable with the repetitive bends as they navigated through them, resulting in higher speeds for bend 4 than bend 2. However, as with the effect of Lap, this did not translate to the post hazard bend (L2B4) which suggests that speed had either reached a ceiling point, or that encountering the hazard on L2B3 negated any further speed increases on L2B4. These findings are illustrated in Figure 6.

**Figure 7:**
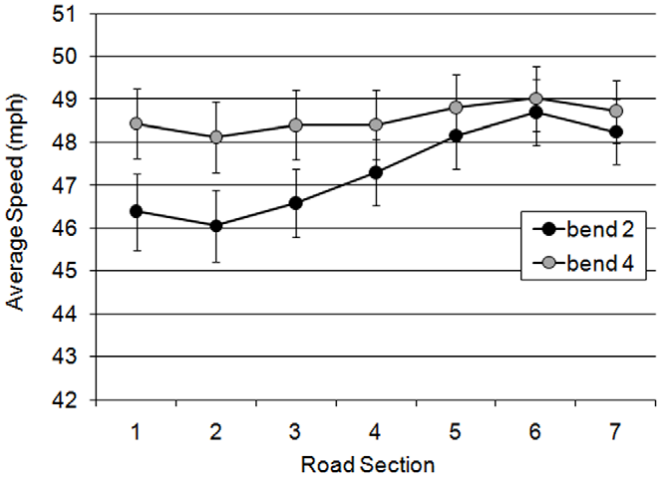
Average speed over 7 bend sections for bend 2 and bend 4 (with standard error bars).

A significant main effect was also observed for road section on mean speed (F(6,348) = 7.464; MSe = 14.431; *p*<0.001) and an interaction between bend and section (F(6,348) = 3.773; MSe = 9.324; *p*<0.01), shown in Figure 7. Simple main effects analysis revealed that there was an effect of section on bend 2 (F(6,360) = 13.824; MSe = 4.759; *p*<0.001), but not on bend 4 (*p*<0.05). Repeated contrasts showed that on bend 2, there was a significant increase in speed through all adjacent sections from 2 to 6 (all *p*<0.05) with the greatest increase between sections 4 and 5 (*p*<0.001). Also, there was a significant decrease in speed between sections 6 and 7 (*p*<0.05). The differences between bend 2 and bend 4 were only significant for the first 3 sections (*p*<0.01). On subsequent sections, average speed on bend 2 resembled average speed on bend 4.

In relation to mean lateral position a significant main effect was observed for rider group (F(2,58) = 23.981; MSe = 0.998; *p*<0.001), illustrating that advanced riders (mean = −5.399 ft) rode closer to the centre line than both novice (mean = −7.331 ft; *p*<0.001) and experienced riders (mean = −7.241 ft; *p*<0.001) during the non-hazard left bends. A significant effect was also observed for lap (F(1,58) = 5.534; MSe = 7.886; *p*<0.05), with riders generally riding closer to the middle of the road on Lap 2 (mean = −6.497 ft) compared to Lap 1 (mean = −6.817 ft). However, these main effects were subsumed by a significant lap × rider group interaction (F(2,58) = 4.424; MSe = 7.886; *p*<0.05), which revealed that the move towards the centre line noted in Lap 2 was only significant for novices (F(1,19) = 11.829; MSe = 0.500; *p*<0.01).

**Figure 8:**
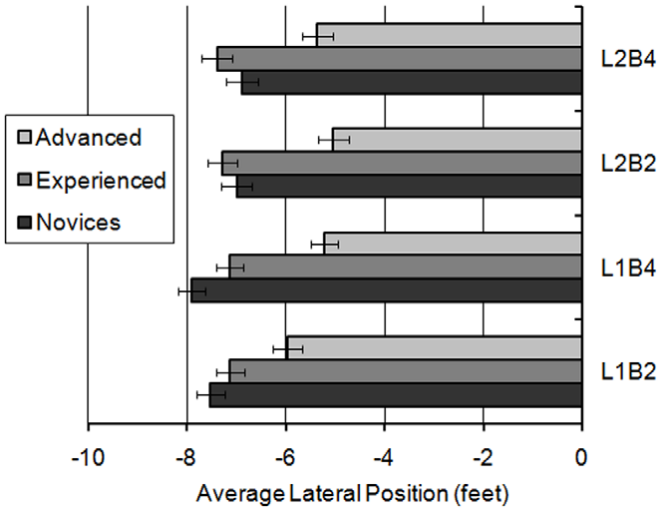
Average lateral position of rider groups for bends 2 and 4, over Lap 1 and 2.

An interaction between rider group × lap × bend also approached significance (F(2,58) = 2.745; MSe = 7.823; *p* = 0.07). Simple main effects analysis revealed that the advanced riders rode significantly closer to the centre than the novice and experienced riders on both bends on both laps (maximum *p*<0.05). Novice riders rode closer to the centre of the road on L2B4 compared to L1B4 (F(1,19) = 8.123; MSe = 1.256; *p*<0.05). There were no effects of lap or bend for experienced riders (*p*<0.05). However, on Lap 1 the advanced riders rode the fourth bend (L1B4) closer to the centre than the second bend (L1B2; F(1,19) = 4.789; MSe = 1.161; *p*<0.05), and also rode the second bend closer to the centre on Lap 2 (L2B2) than on Lap 1 (L1B2; F(1,19) = 9.214; MSe = 0.941; *p*<0.01). These findings are illustrated in Figure 8. There are two interesting points to take from this result: First, the novices appear to move more towards the centre of the road after seeing the hazard. Secondly, advanced riders appeared to use their position on Lap 1 Bend 2, as a gauge for how they approached subsequent bends. After Lap 1 Bend 2 they moved closer to the centre line on all other bends, perhaps reflecting dissatisfaction with their position on this initial bend. The experienced riders do not change their position at all.

The analysis of lateral position also revealed a significant main effect of curve section (F(6,348) = 146.203; MSe = 4.520; *p*<0.001), an interaction between rider group × curve section (F(12,348) = 5.024; MSe = 4.520; *p*<0.001) and an interaction between rider group × curve section × bend (F(12,348) = 1.926; MSe = 2.075; *p*<0.05).

**Figure 9:**
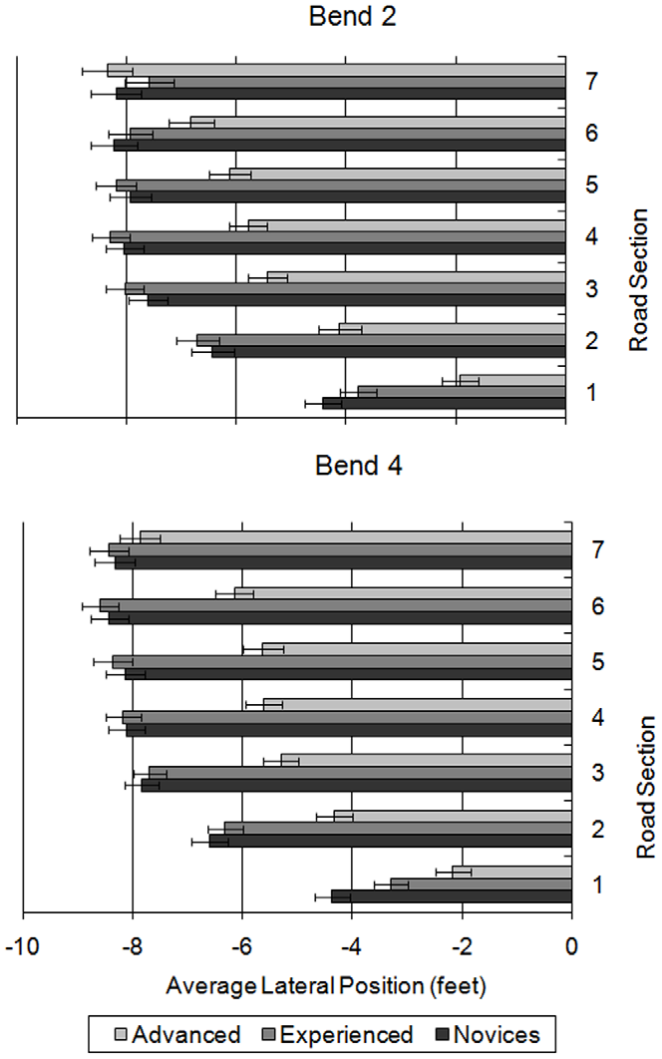
Average lateral position of the rider groups over the seven bend sections for bend 2 (upper panel) and bend 4 (lower panel).

As shown in Figure 9, on Bend 2 novices moved towards the left-hand edge of the road between sections 1 and 4 (all *p*<0.05). On bend 4, the novice riders still moved over to the left at the start of the bend, but only the differences between the first three sections were significant (*p*<0.001). The experienced riders showed a similar pattern, moving over to the left at the start of the bends. However, differences were significant between adjacent sections from 1 to 3 for bend 2 (*p*<0.001) and from 1 to 4 for bend 4 (maximum *p*<0.05). In contrast, the advanced riders continued to move to the left after the apex of the bend, significantly changing their lateral position between all adjacent sections (maximum *p*<0.05), apart from sections 3 to 4 in bend 2 and between sections 3 and 5 in bend 4. Despite this, advanced riders rode significantly closer to the centre line of the bend than the experienced and novice riders during sections 1 to 5 on bend 2 (*p*<0.01) and during sections 1 to 6 on bend 4 (*p*<0.001, apart from advanced vs. experienced in section 1 where *p*<0.05).

If the adoption of a ‘racing line’ is indicated by a move from the centre line across to the left-hand side of the road at some point during a left-hand curve, then it is clear from Figure 9 that experienced and novice riders adopted a more pronounced racing line than advanced riders, and that they initiated the racing line earlier in the bend. The rapid shift in positioning suggests they adopted a racing line before they could see around the blind bend. The advanced riders however adopted a less severe racing line, involving a smaller shift to the left that was spread over a longer distance and time frame. This was exaggerated even further in bend 4 and suggests that advanced riders were aware of the need for visibility over and above the desire for the racing line.

**Figure 10:**
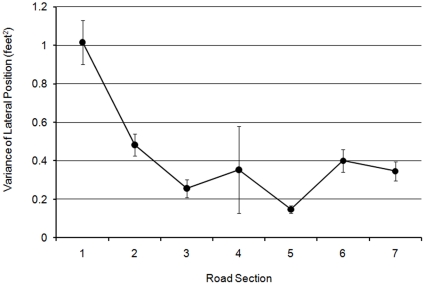
Variance of lateral position over seven bend sections (with standard error bars).

The final analysis of the non-hazard left bends compared the variance of lateral position across rider group, bend, lap, and road section (3×2×2×7). There was a significant main effect of curve section (F(6,348) = 7.825; MSe = 2.443; *p*<0.001). Repeated contrasts revealed significant decreases in variance of lateral position between sections 1 and 2, and between sections 2 and 3, and a significant increase in variance of lateral position between sections 5 and 6 (*p*<0.001). These findings are illustrated in Figure 10. Unlike the analysis of the left-hand hazard bend (L2B3) there were no main effects or interactions involving rider group. This suggests that any group differences in lateral variance across road section on L2B3 were primarily driven by the appearance of the hazard.

### Responses to the right-hand hazard bend

**Figure 11:**
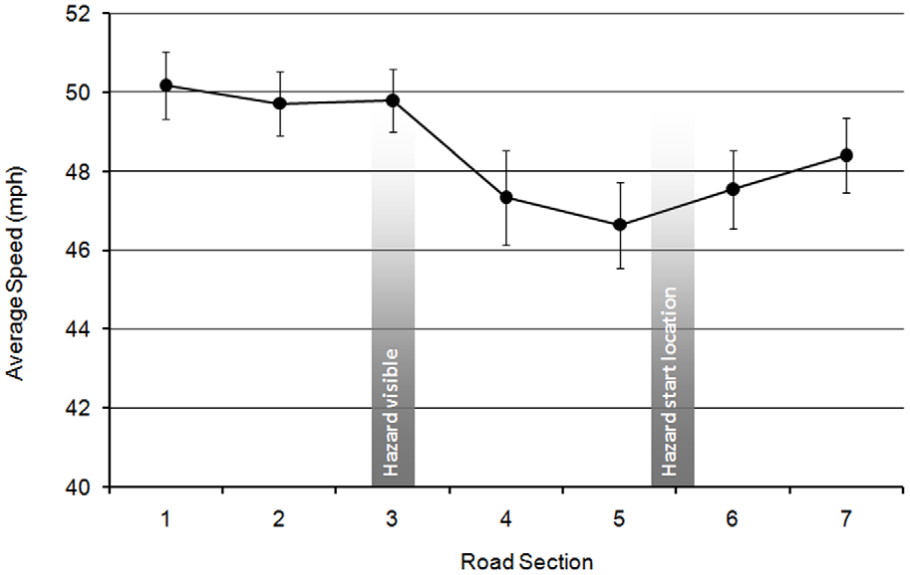
Average speed for different sections of the right-hand hazard bend (with standard error bars).

A series of 3×7 ANOVAs were conducted on data for speed, lateral position, and the variance of lateral position comparing the measures of each rider group across the 7 sections of the curve. Analysis of average speed revealed a significant main effect of curve section (F(6,348) = 6.567; MSe = 18.004; *p*<0.001). Repeated contrasts showed that riders significantly decreased their speed between sections 3 and 4 when the hazard first became visible (*p*<0.01), then significantly increased their speed between sections 6 and 7 (*p*<0.05) after the hazard (Figure 11). There was no effect for rider group and no interaction between rider group and curve section.

The same analysis was conducted on mean lateral position. It should be noted however that the measures have different ramifications when considering right-hand bends rather than left-hand bends. With the left-hand bends a move away from the centre line towards the edge of the road is considered to potentially increase progression (if timed correctly) but may decrease visibility around the bend. With the right-hand bends however, a move towards the centre line, or even over the centre line into the contra-flow lane, will increase progression. Such rightward movement will also decrease visibility around the bend but to a lesser extent than a leftward movement in a left-hand bend. Analysis of the lateral position of the riders revealed a significant main effect of rider group (F(2,58) = 4.140; MSe = 2.53; *p*<0.05) with experienced riders (mean = −4.25 ft) travelling significantly closer to the centre line than advanced riders (mean = −5.55 ft; *p*<0.05). The difference between experienced riders and the novice riders (mean = −5.41 ft) was not statistically significant (*p* = 0.075) despite the novice road position being closer to that of the advanced riders. A significant main effect was also observed for curve section (F(6,348) = 22.487; MSe = 4.747; *p*<0.001) and an interaction between rider group × curve section was identified (F(12,348) = 3.610; MSe = 4.747; *p*<0.001). Repeated interaction contrasts revealed that this interaction was only significant between sections 6 and 7 (*p*<0.05). At section 6, all rider groups were a comparable distance from the centre line (−5.4 ft, −5.2 ft, and −4.8 ft for novice, experienced and advanced riders respectively). In section 7 (the exit spiral) all groups had moved toward the centre line, although the novice and experienced riders made less of a shift than the advanced riders (−3.8 ft, −3.3 ft, and −1.6 ft, respectively). This was supported by simple main effects analysis comparing the three rider groups at each level of road section.

Simple main effects analysis of each rider group across the 7 sections (with repeated contrasts across sections) showed that all three rider groups made significant changes in lateral position over the first three curve sections, moving towards the centre line (*p*<0.01), but did not change their lateral position between sections 3 and 4 as they approached the apex of the bend. Between sections 4 and 5, only novice and experienced riders made a significant change in lateral position away from the centre line in response to the hazard (*p*<0.001). Between sections 5 and 6, only novice and advanced riders made a significant change in lateral position, moving back towards the centre line (*p*<0.05 for novices; *p*<0.001 for advanced riders). Then between sections 6 and 7, all three rider groups made significant changes in lateral position back towards the centre line (*p*<0.001).

**Figure 12:**
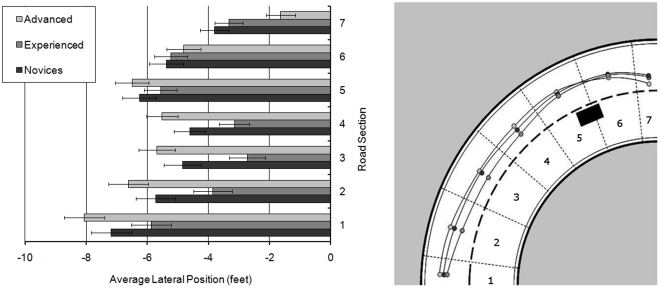
Mean lateral position for different sections of the right-hand hazard bend (with standard error bars).

Simple main effects analysis also showed that in section 1, the effect of rider group only approached significance (*p* = 0.067), with experienced riders riding closer to the centre line than advanced riders while novices were positioned in-between. In sections 2, 3 and 4, the difference between advanced and experienced riders reached significance (maximum *p*<0.05). However, in section 3 the experienced riders were also significantly closer to the centre line than the novice riders (*p*<0.05). In sections 5 and 6, there were no significant differences in the lateral positions of the 3 groups. In section 7, the advanced riders rode significantly closer to the centre line than both the novice riders (*p*<0.01) and the experienced riders (*p*<0.05), supporting the repeating interaction contrasts in identifying the source of the rider group × road section interaction. These results are illustrated in Figure 12.

To summarise, the advanced riders remained further out from the centre line than other riders until they had better visibility through the bend and they had successfully negotiated the hazard. Their safer positioning required less adjustment when the hazard was encountered. In contrast, the experienced riders and, to a much lesser extent, the novice riders shifted their position nearer to the centre line at an earlier point in the bend. When the hazard was encountered, this resulted in a sudden leftward shift away from the centre line.

**Figure 13:**
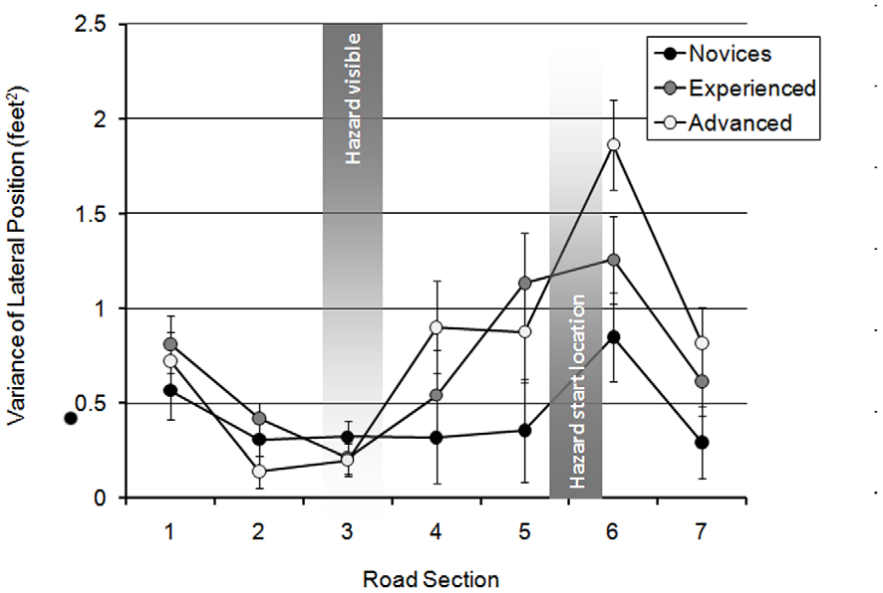
Variance of lateral position for rider groups across the sections of the right-hand hazard bend (with standard error bars).

The final analysis conducted on the right-hand hazard bend was a comparison of the variance in lateral position across rider group and road section. There was a significant main effect of rider group (F(2,58) = 3.636; MSe = 0.197; *p*<0.05) which revealed that advanced riders (mean = 0.79 ft^2^) varied their lateral position more than the novices (mean = 0.43 ft^2^; *p*<0.05). The experienced riders (mean = 0.71 ft^2^), were not significantly different to the other two groups. There was also a main effect of section (F(6,348) = 12.252; MSe = 0.645; *p*<0.001) and a significant interaction between rider group × section (F(12,348) = 1.895; MSe = 0.645; *p*<0.05). Simple main effects analysis revealed that the effect of section was significant for all three rider groups. Novices increased lateral variance between sections 5 and 6 (*p*<0.05) and decreased lateral variance between sections 6 and 7 (*p*<0.05). Simple main effects revealed that the only significant differences between rider groups occurred in section 6, immediately following the initial location of the hazard (F(2,58) = 4.658; MSe = 1.117; *p*<0.05). In this section, advanced riders varied lateral position more than the novice and experienced riders, but only the difference between advanced and novice riders reached statistical significance (*p*<0.05). Therefore, the interaction between rider group × road section mainly stems from the advanced riders increasing lateral movement after the hazard as they move towards the centre line in preparation for next bend. These findings are illustrated in Figure 13.

### Responses to the right-hand non-hazard bends

As with the left-hand non-hazard bends, a series of 3×2×2×7 ANOVAs were conducted on data for speed, lateral position and the variance of lateral position, comparing the three rider groups across Lap 1 and Lap 2, and across the second and fourth bend, for all seven sections of each curve.

**Figure 14:**
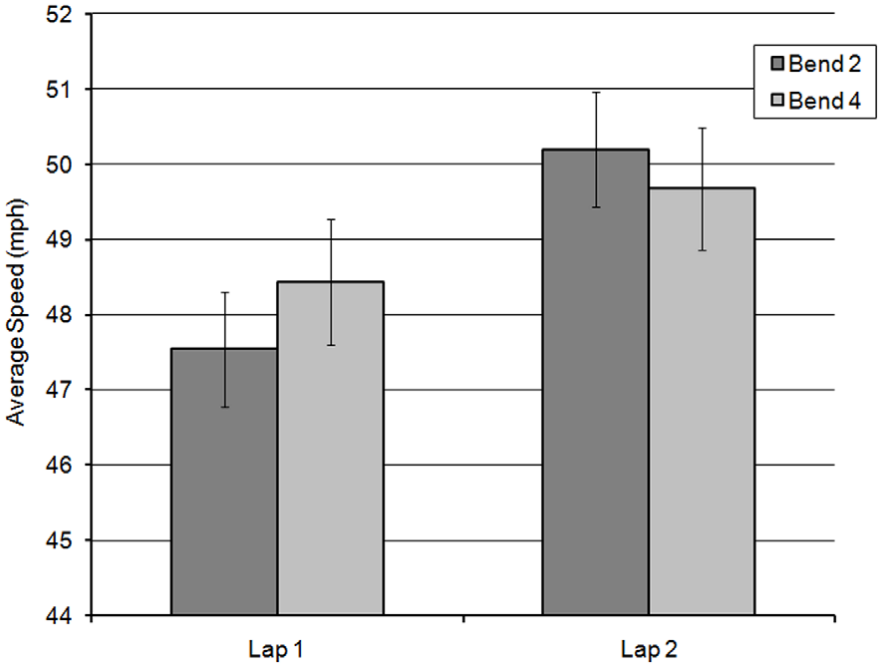
Average speed of riders on the 2nd and 4th right-hand bends across the two laps (with standard error bars added).

Analysis of mean speed revealed a significant main effect of lap (F(1,58) = 11.168; MSe = 145.070; *p*<0.01), which illustrated that riders were faster on Lap 2 (mean = 49.94 mph) than Lap 1 (mean = 47.99 mph). There was also a significant interaction between bend × lap (F(1,58) = 5.234; MSe = 40.622; *p*<0.05), illustrated in Figure 14. While the interaction looks similar to that noted for the left-hand bends (Figure 6), simple main effects showed that riders were significantly faster on both bends in Lap 2, although the effect was greater for bend 2 (F(1,60) = 14.345; MSe = 15.015; *p*<0.001) than for bend 4 (F(1,60) = 4.068; MSe = 11.540; *p*<0.05). Whereas the left-bend hazard ostensibly negated any further increase in speed on L2B4, the right-bend hazard did not fully eliminate the lap effect on bend 4.

**Figure 15:**
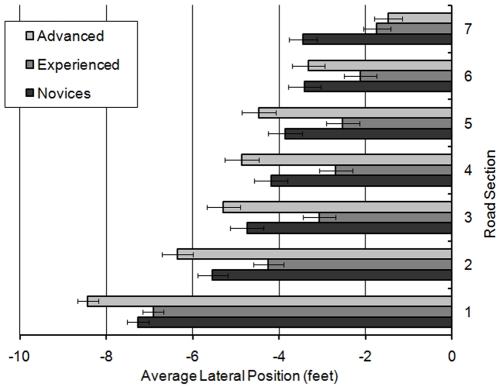
Mean lateral position for rider groups across the right-hand bends (with standard error bars).

The analysis of lateral position revealed a main effect of rider group (F(2,58) = 8.329; MSe = 1.732; *p*<0.01), which showed that experienced riders (mean = −3.328 ft) rode closer to the centre line than advanced riders (mean = −4.885 ft; *p*<0.01) and novice riders (mean = −4.635 ft; *p*<0.05). There was a main effect of lap (F(1,58) = 10.704; MSe = 8.285; *p*<0.01) which revealed that riders were closer to the centre line in Lap 1 (mean = −4.06 ft) than Lap 2 (mean = −4.51 ft). A main effect of section (F(6,348) = 192.168; MSe = 3.890; *p*<0.001) suggested that riders made a significant change in lateral position between all adjacent sections (*p*<0.001), always moving towards the centre line. However, there was also an interaction between rider group × curve section (F(12,348) = 7.204; MSe = 3.890; *p*<0.001) illustrated in Figure 15.

Repeated contrasts revealed that this interaction occurred between sections 1 and 2 (*p*<0.05), 5 and 6 (*p*<0.05), and 6 and 7 (*p*<0.001). Simple main effects analysis showed that it was only the advanced riders who significantly changed lateral position between all adjacent sections (max. *p*<0.05). The novice riders significantly changed their lateral position between all adjacent sections from 1 to 6 (max. *p*<0.05), but did not significantly change lateral position between sections 6 and 7. In contrast, the experienced riders did not significantly change lateral position between sections 4 and 5, but significantly changed lateral position between all other adjacent sections (max. *p*<0.05). In section 1, the advanced riders were significantly further away from the centre line than either the novice (*p*<0.01) and experienced riders (*p*<0.001). However, in sections 2, 3 and 4 the advanced riders no longer differed from the novice riders in terms of lateral position. Both groups however were still further away from the centre line than the experienced riders (for novice vs. experienced *p*<0.05; for advanced vs. experienced max. *p*<0.01). While the experienced riders continued to ride closest to the centre line in section 5, this was only significantly closer than the advanced riders (*p*<0.01). However in section 7, both the advanced and experienced riders were closer to the centre line than the novices (for novices vs. experienced *p*<0.01; for novices vs. advanced *p*<0.001).

In summary, although the novices entered the bend closer to the centre line than the advanced riders, they made less rightward movement than the experienced or advanced riders between the first two bend sections. This means that by section 2, the novices were adopting a similar position to the advanced riders. Advanced riders tended to keep furthest from the centre line until the apex of the bend was passed, at which point they moved towards the centre line. Experienced riders reached this position much earlier having moved significantly towards the centre line before a line of sight was available to them. It appears that the experienced riders were favouring progression over visibility, whereas the advanced riders only positioned themselves for progression once they could see around the bend.

There was also an interaction between bend × section (F(6,348) = 4.267; MSe = 1.262; *p*<0.001), which suggested that riders made an earlier, and more pronounced shift towards the centre line in Bend 4 than in Bend 2 (between sections 1 to 3). After the apex of the bend however, movement towards the centre line was shallower in Bend 4 than that noted in Bend 2.

**Figure 16:**
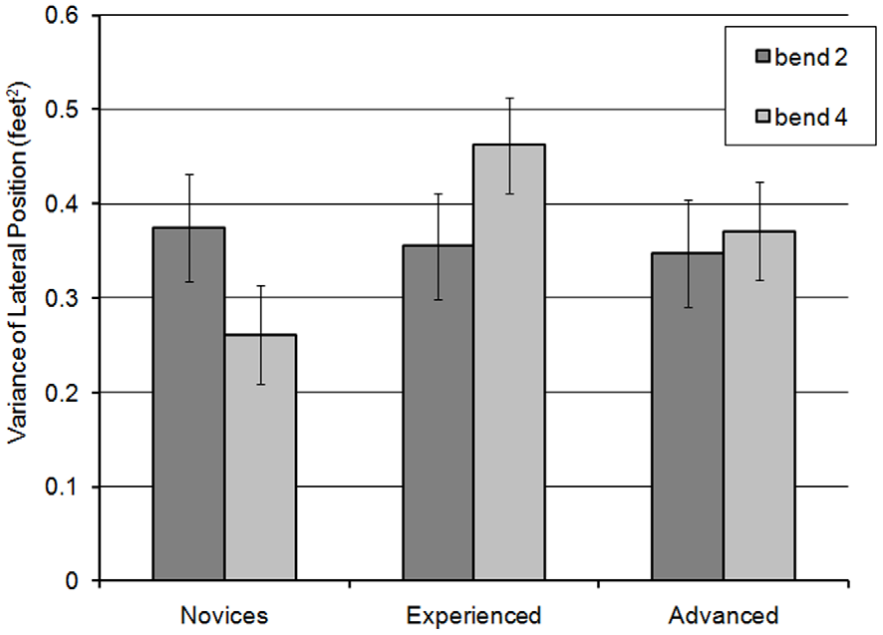
Variance of lateral position for rider group in Bends 2 and 4 (with standard error bars).

The final analysis of the right-hand non-hazard bends was concerned with the variance of lateral position. Although there were no main effects of rider group or bend, there was a significant interaction between the two (F(2,58) = 4.378; MSe = 0.408; *p*<0.05). In Bend 2 all riders varied their lateral position to a similar extent. Simple main effects analysis showed that there was only an effect of rider group for bend 4 (F(2,58) = 3.878; MSe = 0.054; *p*<0.05), with post hoc Scheffé tests revealing that experienced riders varied their position more than the novices (Figure 16).

**Figure 17:**
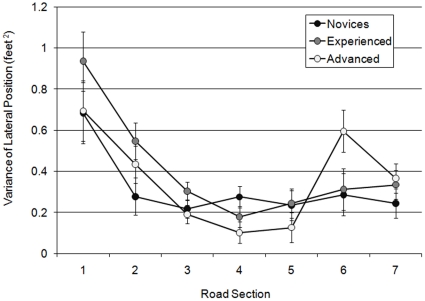
Variance of lateral position for rider groups across the right-hand non-hazard bend sections (with standard error bars added).

There was also a main effect of curve section (F(6,348) = 18.597; MSe = 0.539; *p*<0.001), and a significant interaction between rider group × curve section (F(12,348) = 1.829; MSe = 0.539; *p*<0.05). As illustrated in Figure 17, both experienced and advanced riders significantly reduced lateral variance from sections 3 to 4 (when passing the apex), and then only advanced riders significantly increased lateral variance between sections 6 and 7 (supported by simple main effects analysis, *p*<0.05). In contrast, novices maintained the same variance of lateral position across these sections.

However, curve section also interacted with bend (F(6,348) = 2.598; MSe = 0.253; *p*<0.05) and with lap (F(6,348) = 2.316; MSe = 0.279; *p*<0.05). The first of these interactions was due to increased lateral variance on Bend 4 compared to Bend 2 in the early part of the curve (section 2). The second interaction was due to a general increase in lateral variance on the second Lap immediately following the apex (section 5). As both of these interactions do not involve rider group, and merely describe overall changes in the way that curves are taken with practice, they are not described in any further detail for the sake of brevity.

## Discussion

This study assessed the riding behaviour of novice, experienced and advanced riders navigating bends, both with and without hazards. It was predicted that advanced riders would adopt a more safety-conscious riding style, while novices might adopt a more risky racing line, sacrificing visibility for progression, and requiring more evasive manoeuvres on encountering a hazard. The results however paint a more subtle picture.

In regard to the bends on which the hazards were located (L2B3), all riders adopted similar speeds, with a mean of 50 mph when the hazard first became visible. The first instinct of the riders was to decrease their speed before changing lateral position. Both the left and right bend hazards were initially visible in section 3 of the curve, leading to a significant decrease in speed by the time the riders reached the apex of the bend in section 4. This is clearly a response to the hazard, as no such reduction in speed was noted at the apex of any of the non-hazard bends.

As lateral position only changed significantly by section 5, this suggests that it was a secondary response to the appearance of the hazard. However, analysis of lateral position also revealed group differences. For both the left and right bends, the advanced riders positioned themselves to optimise the balance of progression and visibility. While all riders moved from the outside of the curve towards the inside (indicative of a racing line), the advanced riders chose a shallower racing line which placed them closer to the outside of the curve when the hazard was encountered. This is comparable with the finding that police drivers tended to adopt a more central lane position than civilian drivers in a simulated driving task [26]. As a result, compared with the experienced riders, the advanced group had a significantly smaller shift in lateral position to avoid the left-hand bend hazard, and they did not need to make any significant shift in position to avoid the right-hand bend hazard. On the left hand bend, advanced riders did have to make a slight adjustment to their position to avoid the parked car, but riding any closer to the centre line would have been potentially dangerous if there had been any oncoming traffic. Therefore, advanced riders optimise visibility together with avoidance of collision with oncoming vehicles. Conversely it appears that on both the left and right hazard bends, the experienced riders adopted such early and pronounced racing lines that they required a considerable adjustment to their lateral position in order to avoid the hazards. For some individuals this may have been akin to swerving at the last moment to avoid the unexpected hazard. The hazard was visible at some point during section 3, even to those riders with the most pronounced racing lines. Therefore, while the early racing lines of the experienced riders will have reduced their visibility around the bend, these riders nevertheless managed to reduce their speed by section 4 along with the novices and advanced riders. However, although their initial responses in terms of speed are comparable with the other rider groups, the experienced riders had to reposition themselves more in order to avoid the hazard.

One other outcome of the swerving manoeuvre is that it placed the experienced riders in a sub-optimal location in the exit spiral, where ideally they should have been preparing for the next bend. It appears that the experienced riders (and to a lesser extent, the novice riders) over-compensated slightly when avoiding the hazard, at least on the left-hand hazard bend. Conversely, the better positioning of the advanced riders through the apex of these bends allowed them to take up a more appropriate lane position in the exit spiral in preparation for the subsequent bend. Thus, advanced riders exhibited a safer strategy in the early part of the bend which produced better positioning for progression when exiting the bend.

It is interesting to note however that, in regard to lateral position, the greatest distinction between safe and potentially unsafe riding styles was found in the comparison of the advanced and experienced rider groups rather than between the novice and advanced riders. The novice riders fell in-between the other two groups, behaving more like the experienced riders in the left hazard bend, but behaving more like the advanced riders in the right hazard bend. The novices' behaviour on the right hand bend reflects their recent training: In preparation for the DSA test, novices are taught to move to the left of the lane to increase visibility through the bend. The novices' behaviour on the left hand bend also reflects their recent training to a certain extent: Riders preparing for the DSA test are taught to maintain a more central lane position on left hand bends. Maintaining a central lane position leaves a greater margin for error in regard to avoiding potential collisions with oncoming traffic in the opposite lane, but means that further gains in visibility are sacrificed. This potentially explains why the novices do not move as close to the outside of the curve as the advanced riders, who still maintain a safe distance from any potential oncoming traffic but allow a smaller margin of error. At the apex of the bend, advanced riders are approximately 1 ft to the right of the centre of the lane. However, like the experienced riders, the novices move towards the left hand edge of the lane (approximately 2 ft to the left of the centre of the lane) as they approach the apex of the bend, which means that they have to make a severe adjustment to their lane position when they encounter the parked car.

Since novice riders' positioning was more akin to that of the advanced riders on right hand bends, this might help explain why fewer crashes occur on right hand bends compared to left ones [11]–[13]. While it could be argued that fewer right bend crashes are due to the greater visibility around right hand bends, or simply the fact that most riders are right-handed and may therefore find rightward steering easier to control, the systematic differences between the groups suggest that the majority of non-advanced riders will have a riskier approach in left hand bends than right hand bends.

It is possible that riders base their behaviour on their individual mental models of road hazards. Experience is likely to lead to changes in the rider's mental representation of the probabilities of different hazards occurring (cf. [27]). If a particular type of hazard is encountered frequently, it is more strongly represented in the rider's mental model and it is intuitively perceived as being more likely to occur again. Conversely, if a hazard is encountered infrequently (or not at all), then the rider's mental representation of this hazard diminishes. These mental representations of hazards are also likely to be affected by training, which primes riders to look out for certain types of hazard. Since DSA training of novice riders focuses on possible threats posed by oncoming traffic (i.e. maintaining left positioning on right hand bends and centre-lane positioning on right-hand bends), it is possible that novice riders have an increased representation of the probability of oncoming vehicle hazards in the opposite lane over other types of hazards when negotiating bends. In other words, novices might have been primed (perhaps even *over-primed*) to watch out for oncoming vehicle hazards, but might not have considered a stationary object in their own lane as a potential hazard.

One could also argue that the behaviour of experienced riders represents their own intuitive understanding of the probabilities of such hazards occurring. While encountering an on-coming vehicle very close to the centre line on a blind bend might be more likely to occur than encountering a broken down vehicle, the probability of occurrence is still likely to be relatively low. It is possible that the less cautious behaviour of the experienced riders on the right bend hazards reflects that they have ridden around numerous real life bends without encountering such a situation. Therefore, while training leads to an increase in the representation of right bend hazards and a potential over-estimation of the probability of these hazards occurring, experience leads to a decrease in the representation of these hazards with infrequent exposure. Unfortunately, this means that novices' cautious behaviour on right bends is likely to diminish as increasing experience confirms that encountering oncoming traffic close to the centre line on a blind bend is relatively rare.

The advanced riders however have been ostensibly primed for such low frequency events through their advanced training, thus evoking a shallower racing line that does not sacrifice visibility through the curve. As advanced riders have similar levels of experience (and presumably similar levels of probability estimation) as the experienced group [25], the training has either over-ridden their experience-based estimate of probabilities, or has introduced a completely different consideration such as the severity of the outcome. Certain facts pertaining to the outcome of motorcycle collisions might be sufficient to engender more cautious riding, even though the probability of a particular event could still be only 1∶100,000.

In both the left and right non-hazard bends, similar behavioural patterns were noted across the groups as were found in the hazard bends. For instance, there were no group differences in the speed that riders adopted for non-hazard bends. Speed tended to increase across all non-hazard bends (excluding the final post-hazard bend), suggesting that the more bends the riders encountered, the more comfortable they felt at higher speeds.

As with the hazard bends, group differences on the non-hazard bends only became apparent in the analyses of lateral position. While all riders adopted a racing line, moving from the outside of the curve to the inside, the experienced riders, and to a lesser extent the novice riders, made early shifts of lateral position of significant magnitude. The lateral shift was so extreme that the experienced and novice riders tended to plateau before the apex of the curve, reaching a position that they presumably felt was the closest line they could take. In contrast, the advanced riders made small and continuous shifts through the majority of the curve sections, always remaining further towards the outside of the bend than the other groups through the majority of the curve. By the time the advanced riders had reached the exit spiral however, they had reached the same lateral position as the other two groups, or had moved even further to the inside. It is this safer behaviour in the early part of the bend that provided the advanced riders with the best positioning when they subsequently encountered the hazards.

Encountering a hazard on L2B3 appeared to influence some measures of rider behaviour on the following bend (L2B4). For instance, speed tended to increase from L1B2 to L1B4 and from L1B2 to L2B2 suggesting that practice in negotiating the bends occurred within and between the laps. The bend immediately following the hazard (L2B4) did not follow the same pattern however: instead of showing an increase in speed there was a slight decrease. It is a possibility that the riders had reached a ceiling in regard to choosing a comfortable speed to navigate the bend, or alternatively it might be argued that the failure to increase speed on L2B4 merely reflects the fact that riders had already decreased speed significantly in L2B3 and therefore had not had time to accelerate back up to the higher speeds. We argue against this on the basis that the circuit was designed such that the bends could be taken safely at 55 mph to 60 mph (if the rider was confident enough) and that there was sufficient distance from bend 3 and bend 4 that the motorcycle could enter bend 4 at a higher speed than bend 3. Despite these precautions, it is still possible that the riders reached a subjective ceiling for speed, rather than an objective one.

The lack of group differences in these interactions however renders them less interesting than the impact of the left bend hazard on the lateral position of riders on the subsequent post-hazard bend. The novice riders appeared to reposition themselves further towards the outside of the final left-hand bend after encountering the hazard. Advanced riders did not appear to need to reposition themselves on the final bend following the hazard. Prior to the hazard, the advanced riders had already illustrated that their positioning on the left L1B2 was undesirable, and thus moved further out on the subsequent bend (L1B4). This iterative calibration of their lateral position ensured that they adopted the most appropriate line on the hazard bend. While the novices also reassessed their lateral position, they were presumably insensitive to the more subtle cues picked up by the advanced riders in L1B2, and needed to encounter the more salient hazard before the error of their lateral position became apparent to them. Of most concern however is that the experienced riders did not change their lateral position on the final bend after experiencing the hazard.

Overall the behaviour of the advanced riders fitted our predictions. While they did not differ in speed from the other groups, they took a safer approach to bends with a shallower racing line that optimised vision and progression, resulting in them being in a more appropriate position when the hazard appeared. Furthermore, there is a suggestion that they modified and recalibrated their position on the bends, presumably due to feedback from subtle cues and a continuous improvement strategy which comes from their advanced training. Experts in other domains have been found to be able to predict complex events from subtle cues that might be invisible to the non-expert. Fire-fighters use subtle cues from the movement of smoke to identify the source of a fire, radiologists discern cancer from slight variations in shadow, and presumably, expert motorcyclists can use subtle distinctions in optic flow, visibility and heading to realise that they have missed their optimal line through a curve (see [28] for an overview of non-transport related examples). The novice riders were also sensitive to risk, demonstrating relatively cautious behaviour on right-hand bends and appearing to learn from their positioning errors on the left-hand bends after encountering the hazard. Judging by the performance of the experienced riders however, we should be concerned that the caution adopted by the novice riders will eventually dissipate with increasing experience, and be replaced with a tendency to favour progression over safety. That experienced riders did not modify their approach to the post-hazard bend suggests that their devotion to progression negated the impact of the hazard (even though they had to make the most significant shift in position to avoid it). Current work in our laboratory is assessing whether this might be related to an attribution error in assessing blame for the near collision. Early results do indeed suggest that experienced riders are more likely to externalise blame, which possibly allows them to rationalise away any need to change their own behaviour. In contrast, the advanced riders tended to adopt internal attributions [25].

Hoffman and Fiore [28] argue that expertise requires perceptual relearning. Essentially this means to override previous mappings between visual cues and responses that may have been built up through general exposure and experience, and replace them with new structures that make better use of existing cues, or take advantage of more subtle cues that might not have been apparent previously. This raises the possibility that, in some instances, experienced motorcyclists might develop unsafe associations between certain visual cues and the required behaviour (akin to ‘bad habits’). These perceptual-response mappings would then need to be relearned during the transition to becoming an advanced rider. A similar process has previously been noted in car drivers. Duncan, Williams and Brown [29] compared novice car drivers' performance with experienced and advanced car drivers (with the latter group having taken advanced training with the Institute of Advanced Motorists). On a number of behaviours, they found novice drivers to behave more appropriately than experienced drivers, including mirror checks and appropriate braking on approach to junctions. As with the current study, Duncan et al. found occasions when the advanced group had more in common with the novices than the experienced group. They suggested that such differences tended to occur where negative feedback was unlikely to occur. For instance, while the grinding of gears provides excellent feedback on which to base improvements in changing gear, failing to look in a mirror will only become apparent if a near or actual collision occurs because of the omission. The role of advanced training is therefore to provide the feedback that can only otherwise be obtained at the risk of injury and collision.

This result was mirrored in recent research conducted in our laboratory which assessed how well car drivers spot approaching motorcycles at t-junctions. Eye movement behaviour suggested that experienced car drivers might actually be more susceptible than novices to a “Look But Fail To See” error than novice car drivers [30]. As with the work of Duncan et al. [29], this suggests that over-learned expectancies borne out of years of experience may prove detrimental to road safety in specific situations.

Whilst we would not venture to argue that our sample of novice riders are therefore safer than our sample of experienced riders, it does seem to be the case that they responded more cautiously in some situations. This may, in part, be due to the recent training they had prior to passing their test, or a healthy anxiety of hazards coupled with a lack of confidence in their own abilities. Certainly it appears that the additional training received by our sample of advanced riders has resulted in an overall safer approach to these bends. While one cannot rule out the argument of self-selection in our advanced rider group (i.e. a particular type of motorcyclist might opt for advanced training), it seems plausible that the advanced training has resulted in previously experienced riders overcoming bad habits by relearning the relationship between particular cues and appropriate riding behaviour on bends, therefore making the transition to an expert rider.

### Conclusion

What do the results suggest regarding the causes of crashes on bends that do not involve other vehicles? The Clarke et al. study [5], [6] of police crash reports argues for inappropriate speed or under-steering to be the primary causes of such crashes. However, the current results do not support this. Assuming advanced riders display the safest behaviour, we cannot argue that the other groups adopted an inappropriate speed, as speeds were similar across all groups. Similarly we have no evidence for under-steering in the novice or experienced groups. Instead, an alternative cause is suggested. Our non-advanced riders took a more pronounced racing line through the apex of a bend with constant curvature. If however the bend did not have constant curvature then riders might misjudge the location of the apex (i.e. perceive it be nearer) and thus find themselves drifting back out too soon, or being caught out in a tightening bend by entering too fast. This situation could either lead to the rider running off the curve, or losing control as they suddenly attempt to correct their speed and/or position. Similarly sharper bends may be mis-perceived resulting in an inappropriate racing line, with similar consequences, which might partly explain the greater number of crashes on high-curvature bends [7]–[10].

We should be clear, however, that we are not arguing that speed is not a factor in crashes on bends. Inappropriate speed will certainly contribute to such incidents; it is merely the case that our current sample did not show inappropriate speed (when compared to advanced riders).

One might argue that this suggests that the simulator did not evoke realistic behaviour, as it intuitively seems that speed must be an important factor. However in the absence of any previous literature pertaining to studies of simulated riding in curves, it would be unfair to dismiss the results on the grounds of intuition, or on the basis of accident reports which contain a degree of inference. While it is true that no simulator can perfectly recreate the real world, such ‘face validity’ is less important than ensuring that the particular aspects of the environment that are required for a particular task are adequately represented. For instance, one extremely influential paper that addressed the visual cues required for steering used an Acorn Archimedes computer displaying only three white lines on a black screen (one for the horizon, and two to represent road edges) [31].

While it remains a possibility that the current simulator does not recreate one particularly vital cue to curve negotiation, we argue that it is more likely that the experimental design may account for a failure to identify speed differences. The design of the bends (with high banking and low visibility), plus the fact that each bend was always preceded by an identical bend, may have reduced the variance in speed across the groups. This is not a limitation of the simulation, but a feature of the experimental design. Future research can vary the curvature and visibility through bends, and what road features precede entry into the bend. It is likely that some future conditions may indeed evoke speed differences between groups, though the current demands did not and we have no reason to believe that the simulator is the cause of this null effect. The evidence that points towards speed and under-steering on bends comes from post-crash inferences based on police interviews and crash-site analysis [5], [6], while the current evidence for problems with the racing line comes from direct behavioural observation of a limited sample in a virtual world. While we do not reject the likelihood of speed and under-steering contributing to collisions in certain types of bends, we believe that we have demonstrated that inappropriate lane positioning may also be an important factor.
